# STRUGGLES AND STRENGTH: A QUALITATIVE STUDY OF DIVERSE VOICES FROM THE COVID-19 COPING STUDY

**DOI:** 10.1093/geroni/igac059.1192

**Published:** 2022-12-20

**Authors:** Jessica Finlay, Marisa Eastman, Lindsay Kobayashi

**Affiliations:** University of Michigan, Ann Arbor, Michigan, United States; University of Michigan, Ann Arbor, Michigan, United States; University of Michigan, Ann Arbor, Michigan, United States

## Abstract

This paper aims to explore complex realities and nuanced lived experiences in how diverse older adults are making sense of and dealing with the COVID-19 pandemic. We conducted semi-structured video and phone interviews with 57 COVID-19 Coping Study participants (average age 70.7 years, 44% female, 49% white) from May-July 2021. Qualitative thematic analysis identified physical, mental, social, and economic struggles. These included heightened COVID-19 risk given comorbidities, difficulties accessing healthcare, distressing political events, diminished sense of safety, distance to family and support networks, inability to collectively mourn, and exacerbated financial instability. Coping strategies included exercise, hobbies, spirituality, online activities, engaging with family and friends, and self-care practices. Community-level sources of resilience included vaccines, telemedicine, stimulus checks, and community groups/services. These results highlight profound resiliency and strength to cope with adversities of the pandemic and may inform strategies to support underrepresented and underserved older adults during and after the pandemic.

